# Erinacine A-Enriched *Hericium erinaceus* Mycelium Produces Antidepressant-Like Effects through Modulating BDNF/PI3K/Akt/GSK-3β Signaling in Mice

**DOI:** 10.3390/ijms19020341

**Published:** 2018-01-24

**Authors:** Chun-Hung Chiu, Charng-Cherng Chyau, Chin-Chu Chen, Li-Ya Lee, Wan-Ping Chen, Jia-Ling Liu, Wen-Hsin Lin, Mei-Chin Mong

**Affiliations:** 1 Research Institute of Biotechnology, HungKuang University, Taichung 43302, Taiwan; chchiu@hk.edu.tw (C.-H.C.); ccchyau@sunrise.hk.edu.tw (C.-C.C.); jll.hkn401@gmail.com (J.-L.L.); 2 Bioengineering Center, Grape King Bio Ltd., Taoyuan City 32471, Taiwan; gkbioeng@grapeking.com.tw (C.-C.C.); ly.lee@grapeking.com.tw (L.-Y.L.); wp.chen@grapeking.com.tw (W.-P.C.); 3 Department of Food Science, Nutrition, and Nutraceutical Biotechnology, Shih Chien University, Taipei 10462, Taiwan; 4 Institute of Food Science and Technology, National Taiwan University, Taipei City 10617, Taiwan; 5 Department of Bioscience Technology, Chung Yuan Christian University, Taoyuan City 32023, Taiwan; 6 Institute of Biotechnology, National Changhua University of Education, Changhua County 50007, Taiwan; 7 School of Pharmacy, China Medical University, Taichung 40402, Taiwan; wslin@mail.cmu.edu.tw; 8 Department of Food Nutrition and Health Biotechnology, Asia University, Taichung 41354, Taiwan; 9 Department of Medical Research, China Medical University Hospital, China Medical University, Taichung 40402, Taiwan

**Keywords:** erinacine A, *Hericium erinaceus*, antidepressant, inflammation, monoamines, restraint stress

## Abstract

Antidepressant-like effects of ethanolic extract of *Hericium erinaceus* (HE) mycelium enriched in erinacine A on depressive mice challenged by repeated restraint stress (RS) were examined. HE at 100, 200 or 400 mg/kg body weight/day was orally given to mice for four weeks. After two weeks of HE administration, all mice except the control group went through with 14 days of RS protocol. Stressed mice exhibited various behavioral alterations, such as extending immobility time in the tail suspension test (TST) and forced swimming test (FST), and increasing the number of entries in open arm (POAE) and the time spent in the open arm (PTOA). Moreover, the levels of norepinephrine (NE), dopamine (DA) and serotonin (5-HT) were decreased in the stressed mice, while the levels of interleukin (IL)-6 and tumor necrosis factor (TNF)-α were increased. These changes were significantly inverted by the administration of HE, especially at the dose of 200 or 400 mg/kg body weight/day. Additionally, HE was shown to activate the BDNF/TrkB/PI3K/Akt/GSK-3β pathways and block the NF-κB signals in mice. Taken together, erinacine A-enriched HE mycelium could reverse the depressive-like behavior caused by RS and was accompanied by the modulation of monoamine neurotransmitters as well as pro-inflammatory cytokines, and regulation of BDNF pathways. Therefore, erinacine A-enriched HE mycelium could be an attractive agent for the treatment of depressive disorders.

## 1. Introduction

Depression, a psychiatric disorder characterized by a low self-esteem, altered mood, hopelessness, reduced interest/pleasure in daily activities and persistent thoughts of death or suicide, has become a significant global health issue and economic burden [1]. The lifetime prevalence of depression is approaching 20% of the population and is expected to be the second leading cause of incapacity worldwide by the year 2020 based on data from the World Health Organization [2]. The etiopathology as well as the clear mechanisms underlying depressive disorders are still far from understood, since depression is a highly complicated psychiatric illness. Stress, which can induce neuroinflammation, mitochondrial damage, neuroplastic deficits and intracellular signaling pathways, has been implicated to act as a major determinant for the onset of depression, and may provide a novel target for preventing neurodegeneration [3,4]. The animal models and clinical studies on the link between stress and depressive disorders suggest that antioxidant agents can reduce oxidative stress through scavenging reactive oxygen species (ROS) and reactive nitrogen species (RNS), which further protect against neuronal damage induced by stress [5,6,7,8,9]. In addition, stress-induced depression has been shown to alter the levels of monoamine neurotransmitters such as serotonin (5-HT), along with behavioral changes in animal models [10,11]. Numerous studies reported that normalizing the disturbed monoaminergic neurotransmitters is associated with treating depressive disorders [12,13,14]. Furthermore, a growing body of evidence has demonstrated that stress negatively regulates the level of brain-derived neurotrophic factor (BDNF), which may contribute to the impairment of the dendritic plasticity and hippocampal neurogenesis and be responsible for neuron damage and onset of depression [15,16,17]. Although there are many pharmacotherapies available nowadays, over 30% of depressed patients do not achieve a clinically appreciable improvement with current treatments. The significant limitations of conventional antidepressants include the slow onset for therapeutic actions (weeks to months) and undesirable side-effects such as nausea, diarrhea, migraine headache, sleep disturbance and sexual problems [18,19]. In the view of the impact on depressors, especially for those suicide-risk patients, research focused on the discovery and development of agents with promising efficacy and fewer side effects is urgent.

*Hericium erinaceus* (HE), Houtou mushroom in Chinese, has been used as food and folk medicine in several East-Asia countries for centuries [20]. HE has been documented to display a wide range of beneficial properties, including anticancer, antimicrobial, antihyperglycemic, antioxidant and hypolipidemic activities, and immune modulation [21,22,23,24]. A group of diterpenoids isolated from the cultured mycelia of HE, namely erinacines, were demonstrated to be potential enhancers of nerve growth factor (NGF) biosynthesis in cultured astrocytes [25,26,27]. The increased production of NGF is correlated with proper neural growth and maintenance [28,29]. Importantly, in particular, erinacine A has been reported to exhibit the protective effect against ischemic injury, Parkinson’s and Alzheimer’s diseases in vivo [30,31,32]. Therefore, erinacine A-enriched HE is attracting attention and may serve as a promising agent having neurotrophic activity with potential application in ameliorating neurodegenerative disorders.

Restraint stress (RS) has been extensively applied to induce a depression-like state for screening the effectiveness of antidepressant activities [33]. However, there is no quantitative data regarding the antidepressant-like activities of HE in a repeated RS-induced mouse model of depression. The aim of this present study, thus, was to study the effects of erinacine A-enriched HE mycelium and reveal the possible mechanisms using an RS mouse model. In relation to that, the behavioral alterations and the contents of monoamines, proinflammatory cytokines, and depression-related protein expressions were assessed.

## 2. Results

**Figure 1:**
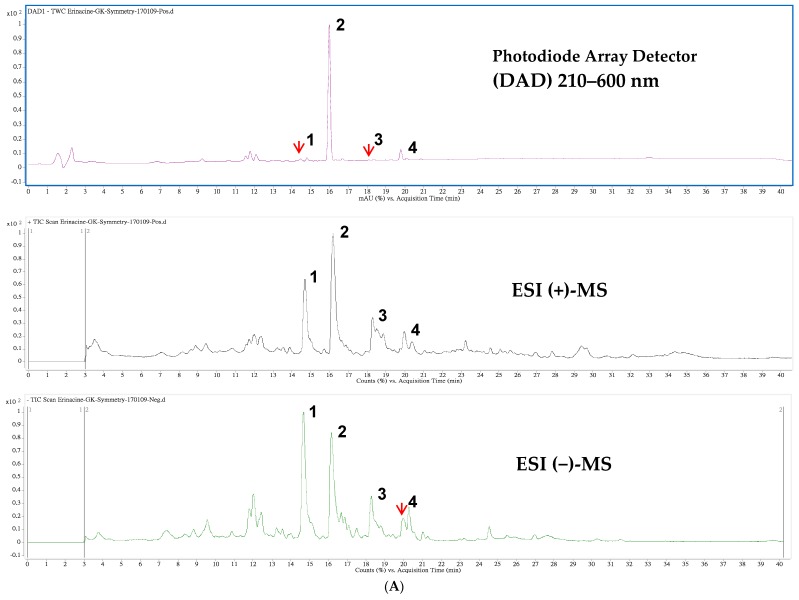

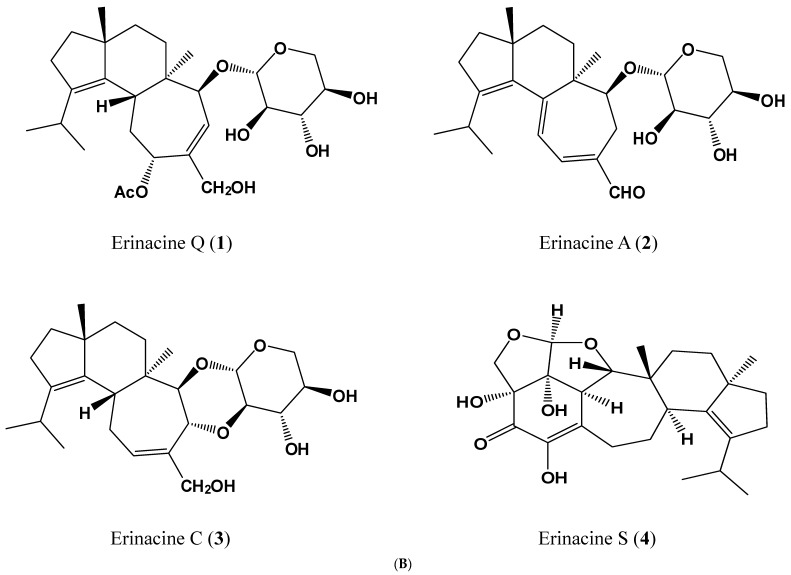
High-performance liquid chromatogram (HPLC) detected at 210–600 nm (**top**), and the total ion chromatograms of positive (**middle**) and negative (**bottom**) ionization mass spectrometry from *Hericium erinaceus* mycelial extracts. The labeled peak numbers were denoted as erinacine Q (**1**), erinacine A (**2**), erinacine C (**3**) and erinacine S (**4**) and are referred to Table 1 (**A**); The chemical structures of four tentatively identified components in the ethanolic extract from mycelia of *H. erinaceus* (**B**).

The chromatograms generated by high-performance liquid chromatogram (HPLC) and liquid-chromatography–electrospray ionization–mass spectrometry (LC–ESI–MS) with positive and negative ionization modes of the ethanolic extract from mycelia of *H. erinaceus* are displayed in Figure 1A. Peak 2 was verified to be erinacine A (**2**) and the other three peaks were tentatively identified comparing to the prepared standards (kindly provided by Dr. CC Chen, HungKuang University, Taichung, Taiwan) as previously reported [32]. The chemical structures and mass spectral characteristics of four major peaks are illustrated and described in Figure 1B and Table 1, respectively. The contents of those peaks were quantified from the established calibration curve as erinacine A (**2**) with the highest amount of 5.0 mg/g dry weight (Table 1).

**Figure 2:**
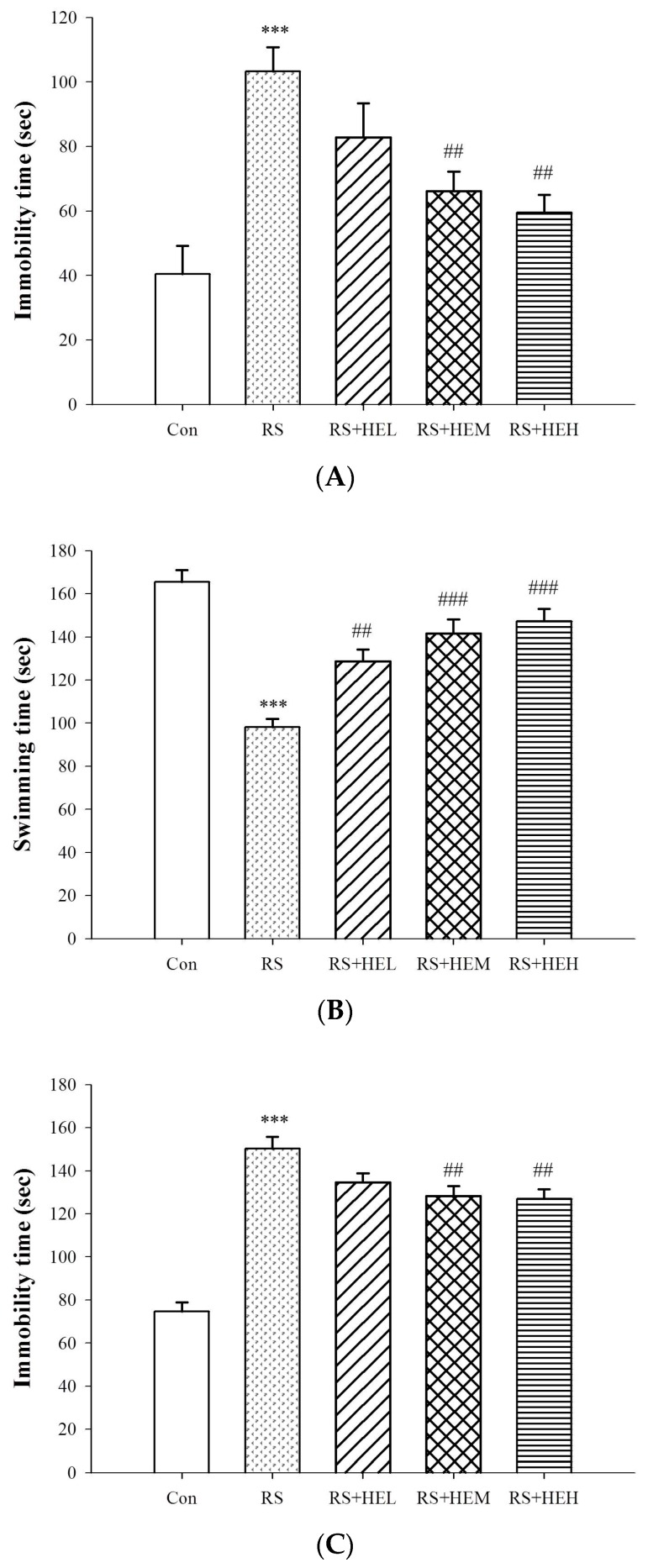
Effects of erinacine A-enriched *Hericium erinaceus* mycelium on immobility time of tail suspension test (TST) (**A**), swimming time of tail suspension test (FST) (**B**), and immobility time of FST (**C**) in repeated restraint-stressed mice. Values are mean ± SEM (*n* = 10 per group). Con: normal control mice, RS: mice received vehicle treatment followed by repeated restraint stress, RS + HEL: mice received low dose of HE (100 mg/kg body weight) treatment followed by repeated restraint stress, RS + HEM: mice received middle dose of HE (200 mg/kg body weight) treatment followed by repeated restraint stress, RS + HEH: mice received high dose of HE (400 mg/kg body weight) treatment followed by repeated restraint stress. *** *p* < 0.001 vs. the Con group; ^##^ *p* < 0.01 and ^###^ *p* < 0.001 vs. RS group. Significant differences between groups were determined using one-way ANOVA and Tukey’s post-hoc test.

To examine the antidepressant-like effect of HE treatment, behavioral responses of the immobility time in the mouse tail suspension test (TST) and forced swimming test (FST) were carried out and are shown in Figure 2A,C, respectively. The results indicated a significant anti-immobility effect elicited by the treatment of HE at the doses of 200 and 400 mg/kg in the TST (*p* < 0.01) and FST (*p* < 0.01) as compared to the vehicle-treated stressed mice (RS group). In addition, HE at the doses of 100, 200 and 400 mg/kg increased the swimming time in the FST (*p* < 0.01, *p* < 0.001 and *p* < 0.001, respectively) as compared to the RS group (Figure 2B).

The ability of HE to modulate emotional reactivity in stressed mice was examined and Table 2 reveals the results of HE on the assayed parameters in the elevated plus maze over the 5-min test. The data showed that there was a significant increase of the number of entries in open arm (POAE) in stressed mice treated with medium and high doses (200 and 400 mg/kg) of HE (*p* < 0.01) as compared to the RS group. The percentage increases in the POAE were 21.1% and 24.1%, respectively. Furthermore, stressed mice treated with HE at 200 and 400 mg/kg significantly increased the time spent in the open arm (PTOA) by 22.4% and 22.1%, respectively, as compared to the RS group. No significant difference in the number of closed-arm entries (CAE) was observed in all groups.

To exclude the changes in behavior observed in TST and FST that were attributed to the false-positive effect, the responses of HE treatment on locomotor activities in mice were tested. Table 3 depicts the mean locomotor responses of testing mice. The administration of vehicle or various doses of HE to repeated restraint-stressed animals did not give rise to any obvious changes in number of crossing and rearing. On the other hand, the RS-alone group showed higher numbers of defecation by ~82% (*p* < 0.01), and middle and high doses of HE reduced defecation significantly by ~27% as compared to the RS group (*p* < 0.05).

**Figure 3:**
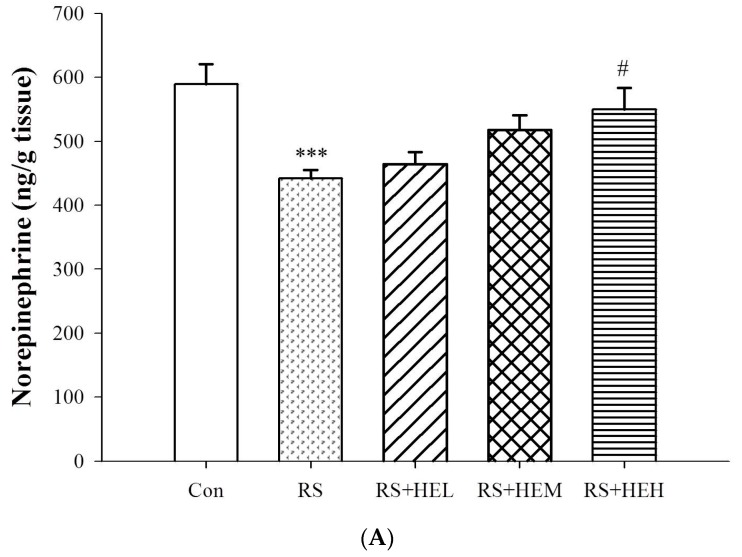

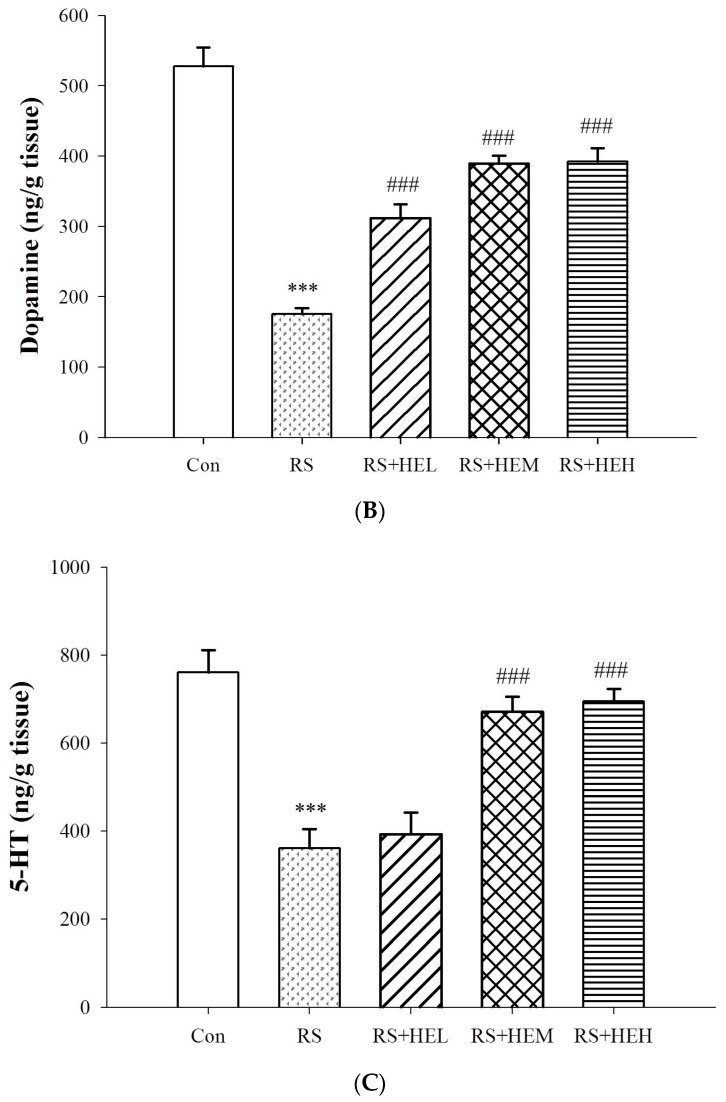
Effects of erinacine A-enriched *Hericium erinaceus* mycelium on norepinephrine (**A**), dopamine (**B**) and 5-hydroxytryptophan (5-HT) (**C**) of hippocampus in repeated restraint-stressed mice. Values are mean ± SEM (*n* = 10 per group). Con: normal control mice, RS: mice received vehicle treatment followed by repeated restraint stress, RS + HEL: mice received low dose of HE (100 mg/kg body weight) treatment followed by repeated restraint stress, RS + HEM: mice received middle dose of HE (200 mg/kg body weight) treatment followed by repeated restraint stress, RS + HEH: mice received high dose of HE (400 mg/kg body weight) treatment followed by repeated restraint stress. *** *p* < 0.001 vs. the Con group; ^#^ *p* < 0.05 and ^###^ *p* < 0.001 vs. RS group. Significant differences between groups were determined using one-way ANOVA and Tukey’s post-hoc test.

The concentrations of norepinephrine (NE), dopamine (DA) and 5-HT were drastically reduced after repeated restraint stress in the vehicle-treated group (RS group) compared with the control group (*p* < 0.001) (Figure 3). Although significant elevation of NE level was found only in high dose of HE treatment (*p* < 0.05), HE (100, 200 and 400 mg/kg) produced profound increases in DA levels in the hippocampal region (*p* < 0.001) as compared to the RS group (Figure 3A,B). Supplementation of medium and high doses of HE helped to revert the stress-induced 5-HT depletion (by raising about 81.6% and 92.5%, respectively) (Figure 3C).

**Figure 4:**
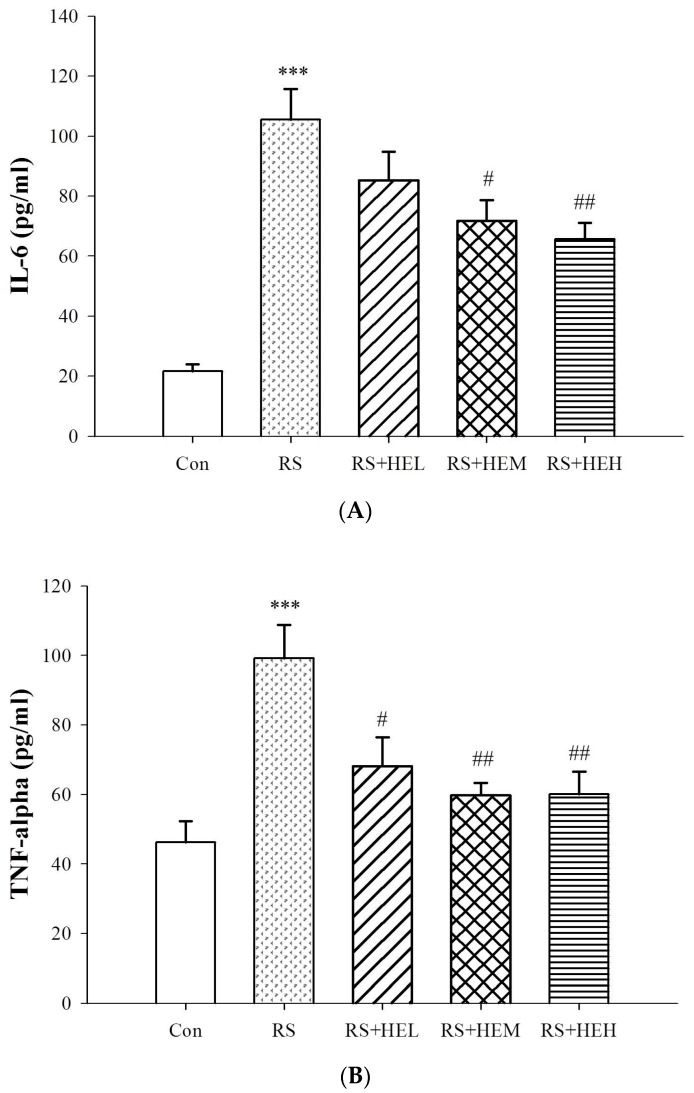
Effects of erinacine A-enriched *Hericium erinaceus* mycelium on plasma IL-6 (**A**) and TNF-α (**B**) levels in repeated restraint-stressed mice. Values are mean ± SEM (*n* = 10 per group). Con: normal control mice, RS: mice received vehicle treatment followed by repeated restraint stress, RS + HEL: mice received low dose of HE (100 mg/kg body weight) treatment followed by repeated restraint stress, RS + HEM: mice received middle dose of HE (200 mg/kg body weight) treatment followed by repeated restraint stress, RS + HEH: mice received high dose of HE (400 mg/kg body weight) treatment followed by repeated restraint stress. *** *p* < 0.001 vs. the Con group; ^#^ *p* < 0.05 and ^##^ *p* < 0.01 vs. RS group. Significant differences between groups were determined using one-way ANOVA and Tukey’s post-hoc test.

Effect of HE on the concentrations of plasma cytokines is illustrated in Figure 4. The levels of interleukin (IL)-6 and tumor necrosis factor (TNF)-α were markedly elevated in repeated restraint stress-treated mice compared with the control group (*p* < 0.001). Supplementation with HE at 200 and 400 mg/kg significantly inhibited stress-induced increases in IL-6 levels (*p* < 0.05 and *p* < 0.01, respectively) and treatment with HE at all doses drastically suppressed plasma TNF-α contents (*p* < 0.05, *p* < 0.01 and *p* < 0.01, respectively) as compared to the RS group.

**Figure 5:**
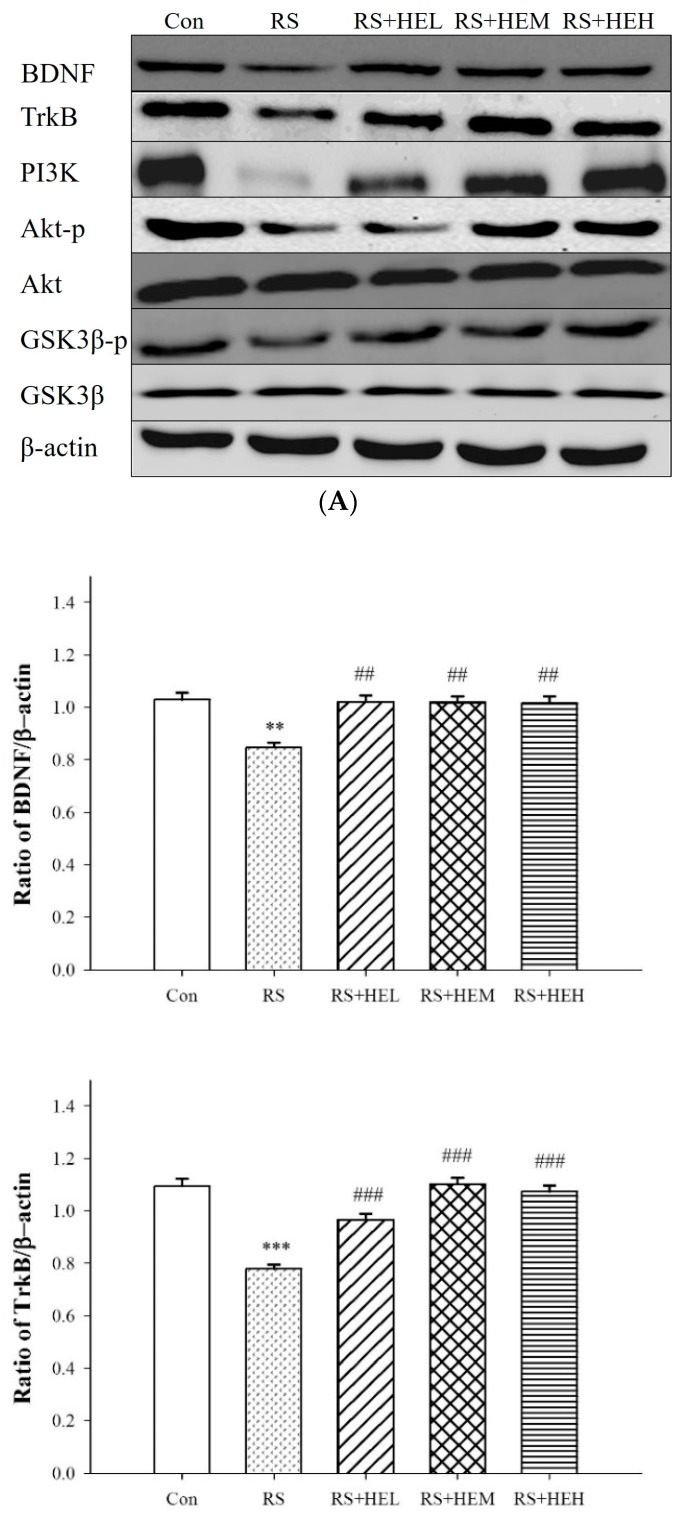

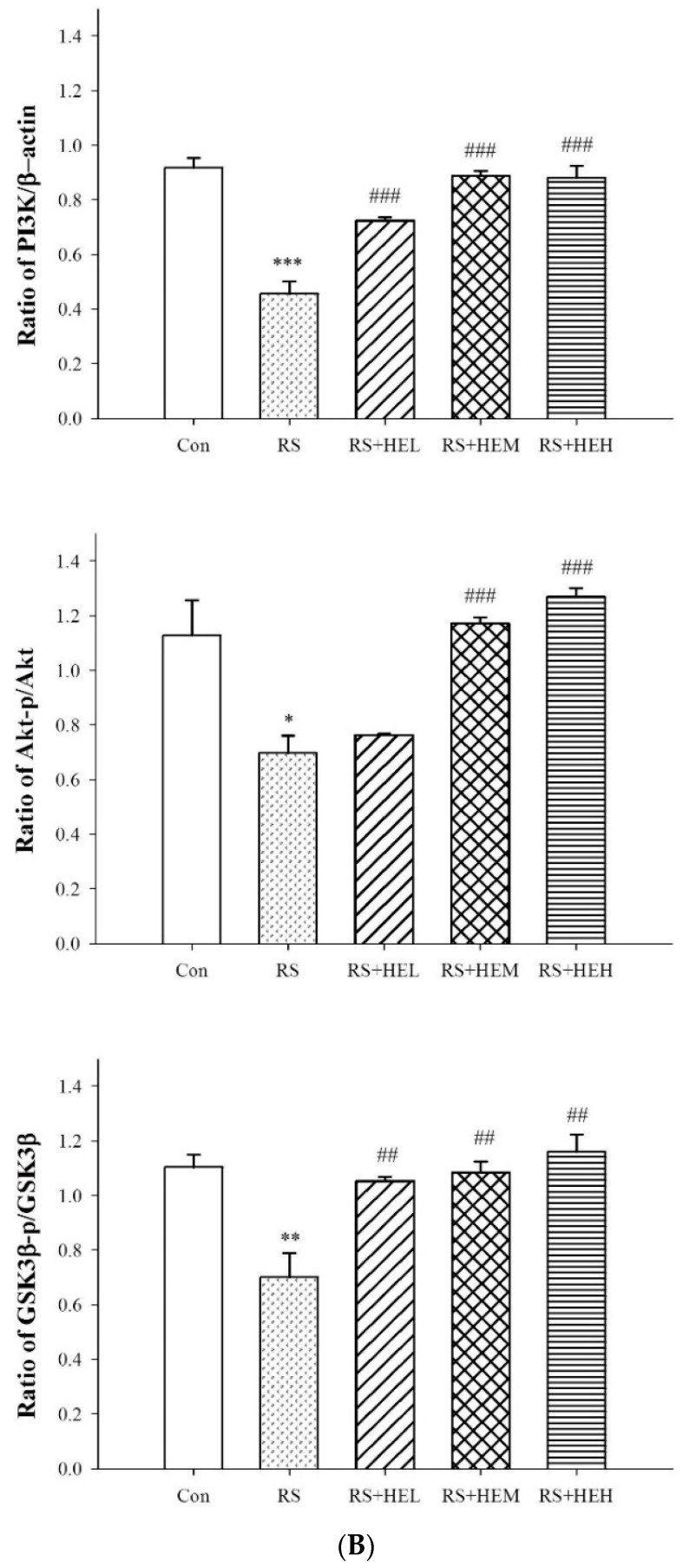
Effects of erinacine A-enriched *Hericium erinaceus* mycelium on hippocampal protein expressions of BDNF, TrkB and PI3K signaling pathways in repeated restraint-stressed mice. (**A**) Western blots of BDNF, TrkB, PI3K signaling pathways and β-actin; (**B**) Densitometric analyses of (**A**), presented as the relative ratio of each protein to β-actin. Values are means ± SEM of three independent experiments. Con: normal control mice, RS: mice received vehicle treatment followed by repeated restraint stress, RS + HEL: mice received low dose of HE (100 mg/kg body weight) treatment followed by repeated restraint stress, RS + HEM: mice received middle dose of HE (200 mg/kg body weight) treatment followed by repeated restraint stress, RS + HEH: mice received high dose of HE (400 mg/kg body weight) treatment followed by repeated restraint stress. * *p* < 0.05; ** *p* < 0.01 and *** *p* < 0.001 vs. the Con group; ^##^ *p* < 0.01 and ^###^ *p* < 0.001 vs. RS group. Significant differences between groups were determined using one-way ANOVA and Tukey’s post-hoc test.

To understand the molecular mechanism underlying the antidepressant-like effect of HE, the expressions of BDNF, TrkB and PI3K signaling pathway proteins with β-actin as control in the hippocampus of mice were examined (Figure 5). Repeated restraint stress decreased the expression levels of BDNF, TrkB and PI3K in the mice brain tissue compared to the control group. HE at tested concentrations was effective to reverse the stress-induced downregulation. Western blotting data revealed that Akt and GSK-3β expressions did not change in all groups. However, repeated restraint stress significantly downregulated Akt-p and GSK-3β-p expressions and the stress-induced decreases in both proteins were prevented by the treatment with HE in the hippocampus of mice.

**Figure 6:**
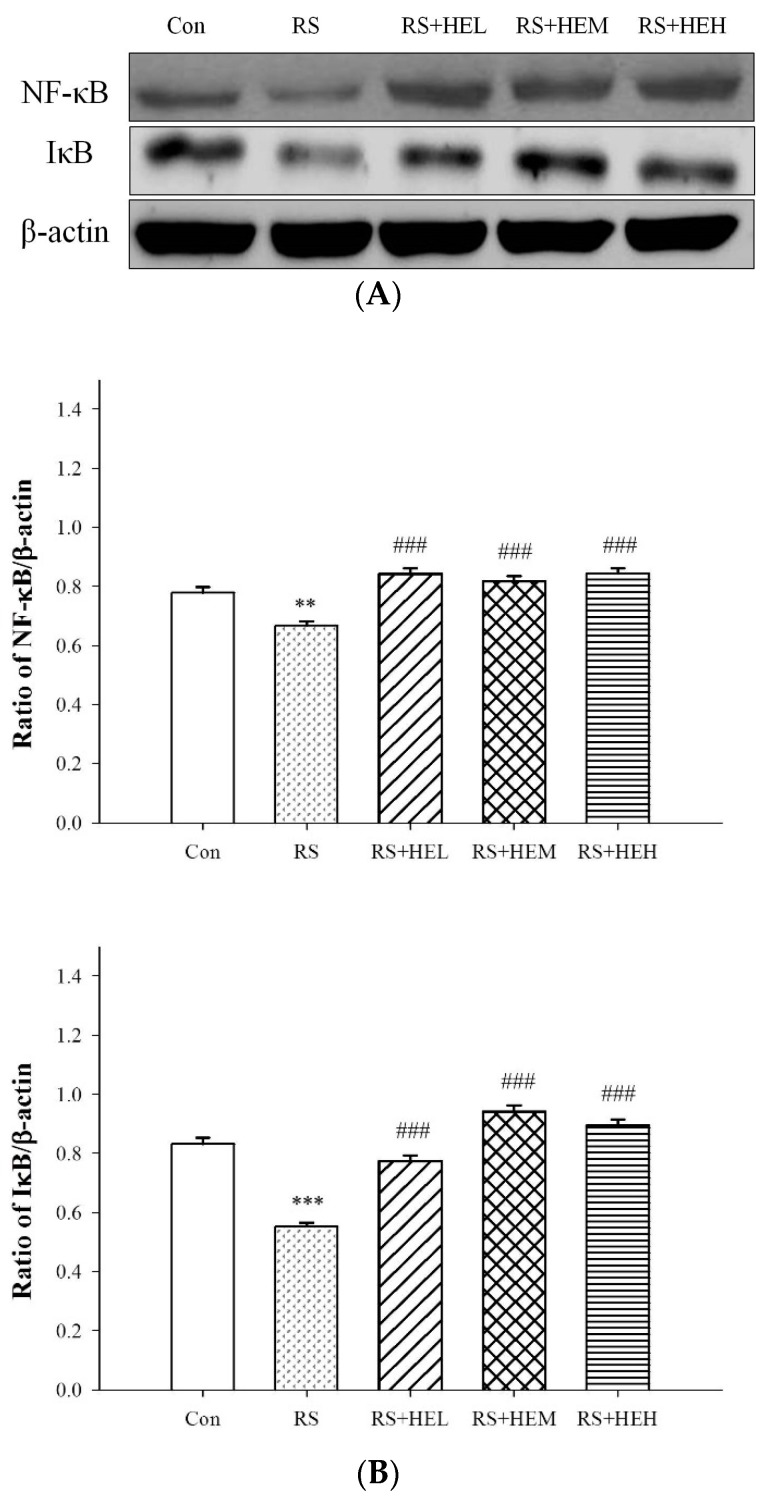
Effects of erinacine A-enriched *Hericium erinaceus* mycelium on hippocampal protein expressions of NF-κB and IκB in repeated restraint-stressed mice. (**A**) Western blots of NF-κB, IκB and β-actin. (**B**) Densitometric analyses of (**A**), presented as the relative ratio of each protein to β-actin. Values are means ± SEM of three independent experiments. Con: normal control mice, RS: mice received vehicle treatment followed by repeated restraint stress, RS + HEL: mice received low dose of HE (100 mg/kg body weight) treatment followed by repeated restraint stress, RS + HEM: mice received middle dose of HE (200 mg/kg body weight) treatment followed by repeated restraint stress, RS + HEH: mice received high dose of HE (400 mg/kg body weight) treatment followed by repeated restraint stress. ** *p* < 0.01 and *** *p* < 0.001 vs. the Con group; ^###^ *p* < 0.001 vs. RS group. Significant differences between groups were determined using one-way ANOVA and Tukey’s post-hoc test.

As a typical signal transduction pathway of pro-inflammatory cytokines, NF-κB and IκB expressions were examined in stressed mice with HE treatment. As shown in Figure 6, significantly lower expressions of NF-κB and IκB in the cytosol fraction of the hippocampus were observed in stressed mice, indicating that the nuclear factor was translocated into nucleus, and enhanced the production of inflammatory mediators. An increasing tendency of both protein expressions could be detected with the treatment of HE, demonstrating that HE could block the NF-κB-induced inflammation, and this was in line with plasma cytokine studies.

## 3. Discussion

Accumulating data suggest that stress plays an important role in the development and manifestation of depression [3,4], and restraint stress (RS) has been applied as a major promoter of depression-like condition to verify the effectiveness of antidepressant activities [33]. The present study investigated the antidepressant-like effects of erinacine A-enriched HE mycelium in the RS mouse model. For the first time, based on the evidence that supplementation of HE decreased immobility times in the mouse TST and FST without affecting the locomotor activity in the mouse open field test (OFT), we have demonstrated that HE exerted remarkable antidepressant-like effects in the RS-induced depressive mice.

Both TST and FST are the most common tools used for evaluating antidepressant potential. In line with other results, mice treated by RS displayed significant immobility in the TST and FST [36,37]. These behaviors were reversed by the treatment of HE (at 200 or 400 mg/kg), indicating an antidepressant-like effect. The antidepressant activity of oral HE treatment was further confirmed by an increase in swimming time analyzed by the FST. Meanwhile, in the OFT, the numbers of crossings and rearings were not altered among groups, indicating that the anti-immobility effects of HE observed in the TST and FST were not attributable to changes in locomotor activities.

Modulating monoamine neurotransmitters, including NE, DA and 5-HT, has been recognized as a major target for elucidating the mechanisms underlying the antidepressant-like effect. The present study demonstrated a significant decrease in the levels of neurotransmitter contents in the hippocampus following 14 days of restraint stress. Our results are in keeping with other studies showing RS induced significantly decreased levels of biogenic amines [38]. Interestingly, HE was effective in restoring these changes in the hippocampus induced by RS following treatment. In the current study, we found that the antidepressant-like effects of HE might stem from increasing the levels of hippocampal NE, DA and 5-HT, which is consistent with previous studies that showed that some botanical extracts mediated the antidepressant-like effect by virtue of an increase of brain monoamines [39,40]. Thus, this result supported the finding that HE administration may lead to antidepressant-like effect by reducing TST and FST immobility time through noradrenergic, dopaminergic and serotonergic modulation in the RS mice. Therefore, we speculated that a possible mechanism underlying the activity is that erinacine A (enriched erinacine in HE constituents) might act as a monoamine neurotransmitter receptor agonist or monoamine neurotransmitter reuptake inhibitor. This possibility needs to be further verified in the future investigation.

There is evidence to suggest that pro-inflammatory cytokines, including IL-1β, IL-6 and TNF-α, contribute to the onset and progression of depressive disorders [41]. Studies have pointed out inflammation could activate some signals which can trigger the transition from inflammation to depression [42]. In fact, increased circulating levels of pro-inflammatory cytokines have been reported with stressed and depressed patients [43,44]. The present study substantiated the enhancement of pro-inflammatory cytokines in the RS depressive mice model. The levels of IL-6 and TNF-α were markedly elevated. Our data are consistent with other reports, which revealed that stressful life events and depressive symptoms are associated with the increase of circulating cytokines in clinical and stress-treated animals [45,46]. Recent studies showed that erinacine A protected from 1-methyl-4-phenyl-1,2,3,6-tetrahydropyridine (MPTP)-induced neurotoxicity as a result of oxidative stress signaling and the JNK/p38/NF-κB pathways in mice [30], and had a protective effect on ischemic myocardial injury via the inhibition of iNOS/p38 mitogen-activated protein kinase (MAPK) and nitrotyrosine in rats [31]. Based on the close link between inflammation and depression, it is reasonable to expect a favorable effect of anti-inflammatory response of erinacine A-enriched HE on depression-like behavior. In fact, our results signified that supplementation with HE drastically inhibited the stress-induced rise of plasma IL-6 and TNF-α contents. Furthermore, HE exhibited antidepressant effects on RS-induced depressive behaviors. Thus, these findings can support the possibility that HE might have an antidepressant effect via regulating the inflammatory response. Although HE shows antidepressant effects via suppressing inflammation, the underlying precise molecular mechanisms remain to be determined. A recent study showed that benzyl alcohol derivatives from *H. erinaceum* attenuate the lipopolysaccharide (LPS)-stimulated inflammatory response through the regulation of NF-κB and AP-1 activity in macrophage cells [47]. The present findings also demonstrated that repeated restraint-stressed animals accompanied by depression-like behavior reduced the expression levels of NF-κB and IκB in the cytosol fraction of hippocampal tissue, and HE-treated mice normalized these levels. It has been believed that NF-κB is a pivotal transcription factor, and it translocates into the nucleus and initiates the transcription of an array of relevant genes (such as pro-inflammatory cytokines) and inducible enzymes (such as inducible nitric oxide synthase (iNOS) and cyclooxygenase (COX)-2 following activation. Accordingly, targeting of the NF-kB pathway is an interesting tactic in the treatment of depression because inflammation plays a critical role in the progression of the disorder [39,40]. In this way, the normalization of NF-κB level could be, at least in part, responsible for the pharmacological effects of HE after repeated restraint stimulation.

It is generally accepted that synaptic plasticity could be influenced by stress condition and the weakening in neuroplasticity might be a key factor in the process of depression [48]. Accordingly, neuroplasticity turns out to be the therapeutic target of antidepressant agents. In this study, the expression of BDNF, the pivotal marker of synaptic plasticity, was examined to reveal the molecular mechanism by which HE drives to normalize depression-like behavior. BDNF is a member of the neurotrophic factor known to participate in the life of neurons during development and to modulate hippocampal-dependent learning and memory [49]. Accumulating evidence supports that BDNF is indispensable for exerting antidepressant effects because it can modulate synaptic efficacy by changing transmitter release and sensitivity [50]. There is also evidence that the lack of BDNF is linked to the pathophysiology of mood disorders [51]. Recently, Wittstein et al. suggested that corallocin C isolated from *Hericium coralloids* is able to induce the mRNA levels of NGF and BDNF for neurite outgrowth of PC12 cells, and the mechanism, at least in part, is connected to act on an upstream target [52]. This is in line with our present study, which found reduced expressions of BDNF and TrkB in the hippocampal region of mice after RS, and that the treatments with HE were effective in restoring the BDNF levels in the brain region. Since the BDNF content was greatly influenced by monoamine transmission [53], the restoration of BDNF content may be an effect of the normalized monoamine content (NE, DA and 5-HT).

Glycogen synthase kinase-3β (GSK-3β) is an enzyme that phosphorylates glycogen synthase, which in turn inhibits glycogen biosynthesis. Moreover, GSK-3β is now believed to play an important role in the pathophysiology of depression and is implicated to be a drug target for the treatment of depression. Furthermore, GSK-3β inhibitors such as thiadiazolidinone NP031115 and AR-A014418 have been reported to be associated with antidepressant effects, as proven by reduced immobility in the forced swimming test [54]. A large amount of evidence has implicated that the pathology of depression might be associated with neuronal inflammation [42]. Literature data indicate that phosphatidylinositol 3-kinase (PI3K) and serine/threonine protein kinase AKT seem to activate immune cells by modulation of the key inflammatory cytokines [55]. In addition, the PI3K/Akt pathway has been reported to play the role as an upstream mechanism of GSK-3β activity regulation, in which Akt might directly phosphorylate GSK-3β, resulting in GSK-3β inactivation. [56]. Irregularities in the PI3K/Akt/GSK-3β pathway are linked in patients with psychiatric illnesses. Therefore, regulation of AKT and GSK-3β may form an important signaling center for depressive therapy. In the present study, we demonstrated that HE was able to increase phosphorylation of Akt and GSK-3β. Altogether, the results presented herein firstly reveal that the antidepressant-like effect of HE involves the activated pathway of PI3K/Akt and inhibition of GSK-3β that converge to increase BDNF.

## 4. Materials and Methods

### 4.1. Cultivation of H. erinaceus

*H. erinaceus*, coded as BCRC 35669, was purchased from the Bioresources Collection and Research Center (BCRC) of Food Industry Research and Development Institute (Hsinchu, Taiwan). The *H. erinaceus* agar slant was transferred and maintained onto a potato dextrose agar plate at 26 °C for 15 days as reported by Li et al. [57]. A piece of mycelium block (20 × 20 mm) was inoculated into a 2-L Erlenmeyer flask containing 1.3 L of modified broth (0.25% yeast extract, 4.5% glucose, 0.5% soybean powder, 0.25% peptone, and 0.05% MgSO_4_; pH was adjusted to 4.5) and the whole broth was incubated at 26 °C on a 120 rpm shaker for 5 days. The fermentation process was then scaled up from a 2-L shake flask to 500-L and 20-ton bioreactors for 5 and 12 days, respectively. Following the fermentation process, the mycelia were harvested by filtration, lyophilized, grounded into powder, weighed and stored in a desiccator at room temperature.

### 4.2. HPLC/ESI–MS Analysis of Hericium erinaceus Mycelial Ethanolic Extract

The HPLC/ESI mass spectrometric analysis of the ethanolic extract of *H. erinaceus* mycelium was accomplished according to a previous report (erinacines Q (**1**), A (**2**), C (**3**), and S (**4**)) [58] with minor modification. In brief, the analysis of extracts was performed using a Waters Symmetry (2.1 × 150 mm, 3.5 µm, Waters Corp., Milford, MA, USA) analysis column fitted with a Security-Guard Ultra C18 guard column (2.1 × 2.0 mm, sub-2 µm, Phenomenex, Inc., Torrance, CA, USA) using an HPLC system consisting of a photodiode-array (PDA) detector. Column temperature was held at 35 °C. The elution solvent system was performed by gradient elution using two solvents: solvent A (water containing 0.1% formic acid) and solvent B (acetonitrile containing 0.1% formic acid). The flow rate during the elution process was set at 0.2 mL/min. A gradient elution was carried out in the first 3 min 30% B, then 30–95% B in 17 min, 95% B isocratic elution for 15 min and finally 95–30% B in 5 min. The absorption spectra of eluted compounds were detected in the range of 210 to 600 nm using the in-line PDA detector monitored at 240, 280, 325, and 340 nm. The compounds having been eluted and separated were further identified with a triple-quadruple mass spectrometer. The system was operated in electrospray ionization (ESI) with both positive and negative ionization modes in a potential of + and −3700 V, respectively applied to the tip of the capillary. Ten μL of sample solution was directly injected into the column using an autosampler. Nitrogen was used as the drying gas at a flow rate of 9 L/min and the nebulizing gas set at a pressure of 35 psi. The drying gas temperature was maintained at 350 °C. The fragmentor voltage was set at 115 V. The separation of ionized mass fragments in the range of 100–800 amu at a scan time of 200 ms/cycle by using quadrupole mass spectrometry. The Mass Hunter software (version: B.01.04; Agilent Technologies, Santa Clara, CA, USA) was applied for all of the data acquisition and manipulation.

### 4.3. Animals and Treatments

Male Institute of Cancer Research (ICR) mice weighing 20–25 g were obtained from the BioLASCO (A Charles River Licensee Corp., Yi-Lan, Taiwan). The animals were housed in regular cages at a constant temperature of 23 ± 2 °C, and relative humidity of 55 ± 5% with 12-h light and dark cycles. They were fed commercial feeds and water ad libitum. The mice were allowed to acclimatize to the laboratory environment for one week before the study. All aspects of this experimental protocol involving animals were evaluated and approved by the Institutional Animal Care and Use Committee of the HungKuang University (10312; 30 September 2014) and were carried out in accordance with the Guidelines for the Care and Use of Laboratory Animals.

The mice were randomly divided into five groups of ten individuals each as follows: unstressed group (Con group), restraint-stressed group (RS group), restraint-stressed plus 100 mg/kg HE-treated group (RS + HEL group), restraint-stressed plus 200 mg/kg HE-treated group (RS + HEM group), and restraint-stressed plus 400 mg/kg HE-treated group (RS + HEH group). HE at three different doses (100, 200, and 400 mg/kg body weight) was given to mice daily by oral administration for 4 weeks, while the control and RS alone groups received the same volume of 0.9% saline. These doses were chosen based on previous reports that demonstrated safety and effectiveness in corresponding disorders [31,32,57]. Starting at the 15th day of the experiment, all mice except Con group were subjected to 14 days of restraint stress. The immobilization procedure was delivered once daily for 2 h by placing animals in well-ventilated transparent restrainers (100 × 40 mm) [59]. After 4 weeks of experiment, multiple behavioral parameters were evaluated followed by biochemical assessments subsequently. 

### 4.4. Behavioral Tests

#### 4.4.1. Tail Suspension Test (TST)

The TST was performed as the method previously described with modifications [60]. Each mouse was suspended by adhesive tape in a metal rod fixed 50 cm above the floor. The trials were videotaped for 5 min, and the total duration of immobility was analyzed by a blinded observer. The mice were considered immobile only when they hung passively and completely motionless.

#### 4.4.2. Forced Swimming Test (FST)

The FST was a modification of a previously described protocol [61]. Animals were individually placed in a glass cylinder (20 cm height × 14 cm diameter) containing 10 cm deep water at 25 ± 2 °C. Each mouse was forced to swim for 5 min and the immobility and swimming times were observed and scored during the trial. The immobility period was defined as the time spent by floating motionless and keeping its head above the water with only necessary movements. Following the test, animals were dried and returned to their cages.

#### 4.4.3. Elevated Plus Maze Test (EPM)

The EPM test was conducted according the method previously described with modifications [62]. The apparatus comprised of two opposite open arms (50 × 9 cm) and two enclosed arms (50 × 9 × 5 cm) which were connected by a common central platform (9 × 9 cm) and elevated to a height of 50 cm above the floor. The floor and the walls of each were wooden and painted black. The mice were placed on the center of the platform facing an open arm and the number of entries and the time spent in both the closed and open arms were videotaped over a 5 min observation period. An entry was considered as all paws of the animal were in the arm. The results were expressed as the percentage of open arm entries (POAE = open arm entries/total arm entries × 100) and the percentage of time spent in the open arms (PTOA = open arm time/total arm time × 100). The apparatus was thoroughly wiped with 70% ethanol before and between tests to avoid any disturbance of residue or odor of the animals.

#### 4.4.4. Open Field Test (OFT)

The open field test was assessed to examine the effect of HE on spontaneous locomotor activity as described previously with modifications [63]. Testing was carried out in an open rectangular acrylic box (60 × 40 × 20 cm) with the floor divided into 96 equal squares (5 × 5 cm). Animals were individually placed in the box and their motor activities were videotaped for a 5 min session. The number of squares crossed with all paws (crossings), of upright posture stood on the hind legs (rearings) and of fecal pellets collected in the box were analyzed by an observer who was unaware of the treatments. The apparatus was thoroughly cleaned with 70% ethanol between tests to remove any residue or odor of the animals.

### 4.5. Determination of Cytokine and Monoamine Neurotransmitter Levels

The mice were sacrificed under CO_2_ anesthesia after completion of behavioral tests. Blood was immediately collected into EDTA tubes and separated in a refrigerated centrifuge at 4 °C and stored at −80°C until use. The total hippocampus was quickly removed, homogenized with ice-cold physiological saline solution and centrifuged at 13,000*g* for 10 min at 4 °C. The supernatant was harvested and reserved at −80°C. The levels of plasma TNF-α, IL-6 and contents of NE, DA and 5-HT in the brain were determined using ELISA kits (R&D Systems, Minneapolis, MN, USA and Novus Biologicals, Littleton, CO, USA, respectively) as described previously [64,65,66] with modifications. Briefly, dispensed protein standards and samples were added to 96-well ELISA plates pre-coated with the capture antibody, followed by the addition of a biotinylated detection antibody and streptavidin conjugated to horseradish peroxidase. The chromogenic reaction was achieved with the addition of tetramethylbenzidine and terminated by using 2 M H_2_SO_4_ after incubation. The absorbance of each well was measured at 450 nm with a VersaMax microplate reader (Molecular Devices, Sunnyvale, CA, USA). In all cases, a standard curve was constructed and the results were quantified from within the curve.

### 4.6. Western Blot Analysis in Hippocampal Tissue 

The whole hippocampus was homogenized with RIPA buffer containing protease and phosphatase inhibitors and protein content was determined by Bio-Rad DC Protein Assay Kit (Bio-Rad, Hercules, CA, USA). Protein lysates were separated by electrophoresis on 12% SDS–PAGE gel and transferred to polyvinylidene difluoride (PVDF) membranes (Bio-Rad) using a semi-dry electroblotting system. After blocking with 5% non-fat milk powder in tris-buffered saline with Tween 20 (TBST), the membranes were incubated with diluted primary antibodies, including BDND, TrkB, PI3K, Akt-p, Akt, GSK-3β-p, GSK-3β, IκB, NF-κB, and β-actin, at 4 °C overnight. After reaction with horseradish peroxidase-conjugated anti-rabbit or anti-mouse immunoglobulin G antibody, the bound-protein bands were visualized by an enhanced chemiluminescence detection system. The relative intensity of proteins of interest was normalized against β-actin.

### 4.7. Statistical Analysis

Statistical analysis was carried out using SPSS version 15.0 for Windows software (SPSS, Chicago, IL, USA). Data were expressed as means ± SEM. Multiple comparisons were analyzed by one-way ANOVA with the Tukey’s post-hoc test. The level of statistical significance was set at *p* < 0.05. 

## 5. Conclusions

In conclusion, HE supplementation normalized the behavioral alterations triggered by restraint stress. The antidepression effect may be attributed to the restoration of hippocampal monoamine neurotransmitters, inhibition of plasma pro-inflammatory cytokines, and modulation of PI3K/Akt/GSK-3β pathway with consequent increase of BDNF expression. All of these pathways are key mechanisms in depression treatment, indicating that HE may represent a potent alternative therapy for depression. The current data confirm that HE may ameliorate altered behavior and neurochemical parameters through several signal transductions, and that these signals may be synchronized with each other.

## Figures and Tables

**Table 1:** Retention time (*t*_R_), UV-Vis and electrospray ionization (ESI) mass spectral characteristics of erinacine A-enriched extract from *Hericium erinaceus* mycelia.

Peak ^1^	*t*_R_ (min)	λ_max_ (nm)	[M + H]^+^	[M + Na]^+^	*m*/*z*	[M − H]^−^	[M + HCOO]^−^	*m*/*z*	MW	Content ^2^	Ref.
**1**	14.47	210		517	285,267		539	529,410	494	0.094	[34]
**2**	15.98	344	433	455	301,283		477	467	432	5.010	[32]
**3**	18.10	222		457	399,417		479	469	434	0.019	[34]
**4**	19.79	284,222		453	431,355	429		409	430	0.374	[35]

^1^ The peak numbers were denoted as erinacine Q (**1**), erinacine A (**2**), erinacine C (**3**) and erinacine S (**4**) and are referred to in Figure 1; ^2^ The content of *Hericium erinaceus* (HE) extract was expressed in mg/g dry weight as mean of three independent analyses.

**Table 2:** Effects of erinacine A-enriched *Hericium erinaceus* mycelial treatment in elevated plus maze test ^1,2^.

	Con	RS	RS + HEL	RS + HEM	RS + HEH
POAE	52.3 ± 2.71	39.4 ± 2.35 **	44.8 ± 2.62	47.7 ± 2.78 ^#^	48.9 ± 2.80 ^#^
PTOA	42.6 ± 2.49	31.7 ± 2.33 **	37.6 ± 2.09	38.8 ± 1.78 ^#^	38.7 ± 2.12 ^#^
CAE	11.1 ± 1.10	13.3 ± 0.53	12.3 ± 0.83	11.0 ± 1.17	11.5 ± 0.73

^1^ Values were obtained from each group of 10 mice and expressed as means ± SEM. Con: normal control mice, RS: mice received vehicle treatment followed by repeated restraint stress. RS + HEL: mice received low dose of HE (100 mg/kg body weight) treatment followed by repeated restraint stress. RS + HEM: mice received middle dose of HE (200 mg/kg body weight) treatment followed by repeated restraint stress. RS + HEH: mice received high dose of HE (400 mg/kg body weight) treatment followed by repeated restraint stress; ^2^ ** *p* < 0.01 vs. normal control; ^#^ *p* < 0.05 vs. RS. Statistically significant difference was analyzed by ANOVA with the Tukey’s post-hoc test.

**Table 3:** The comparison of behavioral indexes in open field test between groups ^1,2^.

	Con	RS	RS + HEL	RS + HEM	RS + HEH
Crossing	35.6 ± 2.68	40.8 ± 2.74	33.6 ± 2.76	35.8 ± 4.82	40.2 ± 3.71
Rearing	24.2 ± 4.02	18.9 ± 1.76	17.9 ± 1.47	19.8 ± 1.88	16.5 ± 2.15
Defecation	6.1 ± 0.93	11.1 ± 0.97 **	10.6 ± 1.04	8.1 ± 0.76 ^#^	8.2 ± 0.70 ^#^

^1^ Values were obtained from each group of 10 mice and expressed as means ± SEM. Con: normal control mice, RS: mice received vehicle treatment followed by repeated restraint stress. RS + HEL: mice received low dose of HE (100 mg/kg body weight) treatment followed by repeated restraint stress. RS + HEM: mice received middle dose of HE (200 mg/kg body weight) treatment followed by repeated restraint stress. RS + HEH: mice received high dose of HE (400 mg/kg body weight) treatment followed by repeated restraint stress; ^2^ ** *p* < 0.01 vs. normal control; ^#^ *p* < 0.05 vs. RS. Statistically significant difference was analyzed by ANOVA with the post-hoc Tukey’s test.
